# Synthesis and Characterization of Clay Polymer Nanocomposites of P(4VP*-co-*AAm) and Their Application for the Removal of Atrazine

**DOI:** 10.3390/polym11040721

**Published:** 2019-04-19

**Authors:** Jorge A. Ramírez-Gómez, Javier Illescas, María del Carmen Díaz-Nava, Claudia Muro-Urista, Sonia Martínez-Gallegos, Ernesto Rivera

**Affiliations:** 1 Tecnológico Nacional de Mexico/Instituto Tecnológico de Toluca, División de Estudios de Posgrado e Investigación, Av. Tecnológico S/N, Col. Agrícola Bellavista, Metepec 52149, Mexico; j.ramirez4499@gmail.com (J.A.R.-G.); cdiazn@toluca.tecnm.mx (M.d.C.D.-N.); cmurou@toluca.tecnm.mx (C.M.-U.); soniakorn@yahoo.com (S.M.-G.); 2 Instituto de Investigaciones en Materiales, Universidad Nacional Autónoma de Mexico, Circuito Exterior S/N, Ciudad Universitaria, Mexico City 04510, Mexico; riverage@unam.mx

**Keywords:** clay–polymer nanocomposites, atrazine, radical polymerization, hexadecyltrimethylammonium bromide, phenyltrimethylammonium chloride, FTIR, TGA, adsorption

## Abstract

Atrazine (ATZ) is an herbicide which is applied to the soil, and its mechanism of action involves the inhibition of photosynthesis. One of its main functions is to control the appearance of weeds in crops, primarily in corn, sorghum, sugar cane, and wheat; however, it is very toxic for numerous species, including humans. Therefore, this work deals with the adsorption of ATZ from aqueous solutions using nanocomposite materials, synthesized with two different types of organo-modified clays. Those were obtained by the free radical polymerization of 4-vinylpyridine (4VP) and acrylamide (AAm) in different stoichiometric ratios, using tetrabutylphosphonium persulfate (TBPPS) as a radical initiator and *N*,*N*′-methylenebisacrylamide (BIS) as cross-linking agent. The structural, morphological, and textural characteristics of clays, copolymers, and nanocomposites were determined through different analytical and instrumental techniques, i.e., X-ray diffraction (XRD), Fourier-transform infrared spectroscopy (FTIR), and thermogravimetric analysis (TGA). Adsorption kinetics experiments of ATZ were determined with the modified and synthesized materials, and the effect of the ratio between 4VP and AAm moieties on the removal capacities of the obtained nanocomposites was evaluated. Finally, from these sets of experiments, it was demonstrated that the synthesized nanocomposites with higher molar fractions of 4VP obtained the highest removal percentages of ATZ.

## 1. Introduction

**Figure 1:**
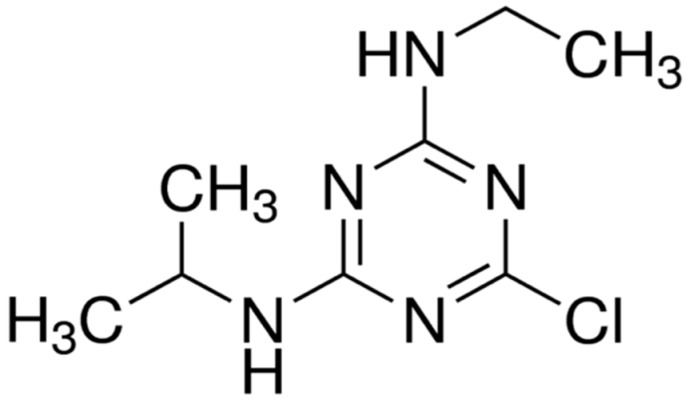
Structure of the atrazine (ATZ) herbicide.

Atrazine (ATZ) is an herbicide that belongs to the family of triazines; its International Union of Pure and Applied Chemistry (IUPAC) name is 6-chloro-*N*-ethyl-*N*-(1-methylethyl)-1,3,5-triazine-2,4-diamine (Figure 1). It is a selective herbicide that is applied to the soil; mainly, its action mechanism involves the inhibition of photosynthesis. Moreover, in plants, it is absorbed through the roots or leaves and it is applied before or after the germination of seeds. One of the main applications of ATZ is for controlling the occurrence of broadleaf and grassy weeds [1,2]. Additionally, ATZ is also known as an endocrine disruptor; this term defines a diverse and heterogeneous set of chemical compounds capable of altering the hormonal balance.

There are many factors that contribute to the pollution of water sources destined to human consumption, through runoff with ATZ. Some of the properties that determine the mobility of ATZ and its metabolites in the environment are their low affinity to the soil components and their high persistence. Moreover, the relative “high” solubility of ATZ in water is the most frequent cause of finding it in surface and underground water bodies [3,4]. The Environmental Protection Agency of the United States (EPA) reported that this herbicide is toxic to numerous aquatic and reptile species, at levels of up to two parts per billion (ppb). Moreover, the EPA recommendation for the treatment of drinking water, polluted with triazines, is filtration with granular activated carbon (GAC) [5].

The degradation of ATZ can be carried out by both biological and chemical reactions. Biological degradation occurs through the activity of microorganisms and it is considered as the main process by which this herbicide is transformed [6,7,8,9]. However, the degradation of ATZ by means of microorganisms is not the most appropriate technology due to the formation of its metabolites, which are more toxic than the herbicide itself. Meanwhile, the chemical degradation of ATZ is carried out mainly by two processes: hydrolysis and photolysis. Hydrolysis commonly leads to the production of hydroxylated compounds such as hydroxyatrazine, desethylhydroxyatrazine, and deisopropylhydroxyatrazine, each one with variable persistence and toxicity [10].

One alternative method employed for the removal of ATZ is the adsorption process through the development of novel adsorbent materials, which is widely used. Some advantages of this method are (i) effectiveness, since it can reduce ATZ concentration to prevent the formation of a metabolite of the herbicide; (ii) simplicity, because its application is the use of columns through which water is passed; (iii) environmentally friendly, thanks to the use of small amounts of adsorbents that can be regenerated; and (iv) economical, because materials are cheaper than those used in other methods. One example of these kinds of materials involves clays modified with a cationic surfactant, also called organo-modified clays, because the nature of the hydrophilic clay is modified by organic cations to form a richer organophilic surface, which confers them a great affinity for organic compounds, being able to remove them from water bodies [11,12,13,14].

These organo-modified clays are incorporated into polymer matrices to obtain clay polymer nanocomposites that attracted attention as adsorbents, since they can be reusable and have a high retention capacity [15,16,17]. It is noteworthy that a nanocomposite consists of at least two main components that are chemically distinct and insoluble. The first of them is the matrix that serves as the continuous phase and could be a polymeric, a metallic, or a ceramic material. The second one is the filler, in the nanoscale range, i.e., from 1 to 100 nm, whose main function is to reinforce the matrix; some examples of fillers are graphene, dichalcogenide materials like MoS_2_ MoSe_2_, WS_2_, or MoTe_2_, or natural materials such as clays or zeolites. Specifically, in our research group, we synthesized different types of clay polymer nanocomposites for different purposes, i.e., for the removal from aqueous solutions of azo dyes [18,19], a triarylmethane dye [20], or even phenolic compounds [21].

The compound 4-vinylpyridine (4VP) is a weak base that, when protonated, can increase its volume due to the incorporation of solvent and electrostatic repulsion between the charged sites. Moreover, the presence of the pyridine ring offers the possibility of anchoring different species, making it a good adsorbent [22]. Acrylamide (AAm) has two reactive centers: the amide group carries out the characteristic reactions of an aliphatic amide and also has weakly acidic and basic properties [23,24]. The double bond of AAm is deficient in electrons and produces Michael-type addition reactions, many of which are reversible. AAm is used in the treatment of water to flocculate solids. On the other side, sepiolite is a raw clay mineral that is used for its organo-modification. This is because of the enhancement in the mechanical properties that it offers to any polymer matrix and because it is very easy to modify with a cationic surfactant [25,26,27].

In this work the organo-modification of a raw clay mineral, consisting mainly of sepiolite, and its nanocomposites, comprising monomers 4VP and AAm in different percentages, were synthesized and characterized. For this purpose, a raw clay mineral, a sepiolite, from Puebla State, in Mexico, was organo-modified with two different cationic surfactants, namely, hexadecyltrimethylammonium bromide (HDTMA-Br) or phenyltrimethylammonium chloride (PTMA-Cl). Afterwards, these organo-modified clays were incorporated into the copolymer structures, p(4VP*-co-*AAm), to obtain two different series of nanocomposites. At last, their morphology, their thermal properties, their spectroscopic characteristics, and their ATZ kinetics adsorption were gauged.

## 2. Materials and Methods

### 2.1. Materials

All the chemicals and materials used in the synthesis and characterization of nanocomposites, as well as those for the ATZ adsorption tests, were used as received without any further purification, unless otherwise stated. Firstly, 4-vinylpyridine (4VP; (FW) = 105.14 g∙mol^−1^, b.p. = 62–65 °C) and acrylamide (AAm; FW = 71.08 g∙mol^−1^, m.p, = 82–86 °C) monomers, *N*,*N*’-methylenebis(acrylamide) (BIS; FW = 154.17 g∙mol^−1^, m.p. ≥ 300 °C) cross-linking agent, hexadecyltrimethylammonium bromide (HDTMA-Br; FW = 364.45 g∙mol^−1^, m.p. = 212 °C) and phenyltrimethylammonium chloride (PTMA-Cl; FW = 171.67 g∙mol^−1^, m.p. = 246–248 °C) cationic surfactants, and formamide (FW = 45.04 g∙mol^−1^, b.p. = 210 °C) solvent were obtained from Sigma-Aldrich. Sodium chloride (NaCl, FW = 74.55 g∙mol^−1^, *T*_m_ = 776 °C) was obtained from JT Baker (Mexico City, Mexico). The ionic-liquid radical initiator of the polymerization, tetrabutylphosphonium persulfate (TBPPS; FW = 710 g∙mol^−1^), was synthesized according to the procedure previously reported elsewhere [28]. Atrazine (ATZ, 6-chloro-*N*-2-ethyl-*N*-4-isopropyl-1,3,5-triazine-2,4-diamine ≥90%) was kindly supplied by Servicios Tecnológicos para la Agrigultura S.A. de C.V. (Irapuato, Mexico) in its commercial form “Calibre 90 DF”. Finally, a raw clay mineral, with sepiolite, supplied by Zeolitech S.A. de C.V. (Puebla, Mexico) and identified by the supplier as A1, was employed.

### 2.2. Raw Clay Mineral Organo-Modification and Cationic Exchange Capacity (CEC)

Firstly, the raw clay mineral (sepiolite) was milled and sieved until it was obtained with a particle size lesser than 44 μm (325 mesh); subsequently, it was stored in plastic bottles free of moisture until it was used. Afterward, 50 g of this clay was put in contact with 500 mL of a NaCl solution (0.1 M) for 3 h with reflux. Next, phases were separated and the clay was decanted; then, another 500 mL of NaCl solution was added, repeating the same procedure until it completed 6 h of reflux. At the end of the total reflux time, the clay was allowed to cool to room temperature and the solution was decanted. After its homoionization, the clay material was washed with deionized water for the elimination of chloride ions, which was verified using silver nitrate (AgNO_3_). In addition, CEC of the clay material was performed as reported by the American Petroleum Institute, API [29,30]. Briefly, 1 g of the raw clay material, 10 mL of distilled water, 15 mL of hydrogen peroxide (H_2_O_2_; 3% *v*/*v*), and 0.5 mL of a sulfuric acid (H_2_SO_4_) solution (0.5 N) were mixed. Subsequently, the mixture was heated and allowed to boil for 10 min. After this time, distilled water was added until a volume of 50 mL was obtained; finally, it was titrated with a methylene blue solution of 0.01 mEq.

### 2.3. Modification of the Homoionized Clay Mineral with HDTMA-Br and PTMA-Cl

The modification of the homoionized clay mineral was carried out with two different cationic surfactants, namely, HDTMA-Br and PTMA-Cl. In the case of the modification of the clay mineral with HDTMA-Br, it was carried out as reported by Hernández-Hernández et al. [21]. Specifically, 10 g of clay material was put in contact with 100 mL of a solution of HDTMA-Br (0.03 M, 30 mEq∙L^−1^) for 48 h at 30 °C and 100 rpm. In the case of the modification with PTMA-Cl, 10 g of clay material were put in contact with 100 mL of a 0.03 M PTMA-Cl solution (30 mEq∙L^−1^), applying the same conditions of temperature and contact time. The concentration of the used surfactant was in concordance with the CEC that was calculated for the raw clay mineral. At the end of this time, the two organo-modified clay minerals, with HDTMA-Br (OMH) or with PTMA-Cl (OMP), were washed with deionized water until the complete removal of bromide or chloride ions, respectively.

### 2.4. Synthesis of the Organo-Modified Clay Nanocomposites (CC)

Organo-modified clay nanocomposites (CC) were obtained according to the following procedure: appropriate amounts of 4VP and AAm comonomers, OMH or OMP organo-modified clays, BIS cross-linking agent, TBPPS polymerization initiator, and a solvent mixture consisting of formamide and deionized water (50% *v*/*v*) were added to a polymerization reactor (Table 1). In all cases, the total amount of the two comonomers (0.1 mol), initiator (TBPPS, 0.25 mol. % referred to the total molar amount of the two comonomers), cross-linking agent (BIS, 3 mol. % referred to the total molar amount of the two comonomers), and the solvent mixture (2 mL) were kept constant. Then, mixtures without TBPPS to avoid spontaneous polymerization were placed in an ultrasonic bath for 3 h at 100 rpm to exfoliate and disperse the clay [17]. After, TBPPS initiator was added and the polymerization reactors were again ultrasonicated until TBPPS was totally dissolved. Finally, nitrogen was bubbled for 30 min and reactors were sealed and placed in an oil mineral bath at 55 °C for 24 h. After polymerization, all samples were extracted and washed with deionized water for several days to remove the solvent mixture and all reagents that did not react.

### 2.5. Swelling Behavior

Equilibrium swelling behaviors were recorded for the synthesized dry materials, one for each stoichiometric ratio, and their initial weight (Po) was recorded. Dry disc samples were immersed in 50 mL of deionized water at room temperature. Weights of swollen discs were measured at different time intervals after excess surface water was removed. This procedure was repeated until there was no weight change. The swelling percentage was calculated according to the following equation:

Sw (%) = [(*W*_s_ − *W*_d_)/*W*_d_] × 100, (1)

where *W*_d_ and *W*_s_ are the initial dried disc sample and the final swollen disc sample weights after a certain period of time, respectively.

On the other side, the critical pH points of the samples were studied while they were immersed in distilled water for 8 h, at room temperature in solutions of pH ranging from 2.2 to 11. In all cases, the inflexion point of the swelling as a function of pH gives us the critical pH point.

Finally, the pH sensitivity was defined from the following equation:

(pH)_s_ = *W*_3_/*W*_7_, (2)

where *W*_3_ and *W*_7_ are the swollen weights of the samples at pH = 3 and 7, obtained after 8 h of swelling.

### 2.6. Characterization

#### 2.6.1. Raw Clay Mineral, Organo-Modified Clay Materials, and CC

X-ray diffraction (XRD) of both the raw clay mineral and the organo-modified clay materials was obtained on a Rigaku X-ray diffractometer to identify the main crystalline phases that compose them. The analyses were performed by the powder method, running the samples from 2° to 85° in 2θ and with a step size of 0.02°.

FTIR spectra for all samples were recorded on an infrared absorption spectrophotometer with Fourier transform (FTIR Varian 640-IR) equipped with a diamond ATR (attenuated total reflectance) accessory. The absorption was measured in a wavenumber range between 4000 and 600 cm^−1^, with a resolution of 16 cm^−1^ and 16 scans.

The morphology of the clay minerals and CC was studied by means of scanning electron microscopy (SEM) using a scanning electron microscope SEM JEOL model JSM 5900LV (Mexico City, Mexico).

In the case of the thermal analysis, the thermal stability of the samples was studied through the thermogravimetric analysis (TGA) in a thermogravimetric analyzer, TA Instruments, model Q5000 TGA (Mexico City, Mexico) under N_2_ atmosphere, from 25 to 850 °C, at a heating rate of 10 °C∙min^−1^. Moreover, differential scanning calorimetry (DSC), using a differential scanning calorimeter, TA Instruments model 2910 (Mexico City, Mexico), was employed in order to determine the thermal transitions of the polymeric materials. For each sample, two consecutive scans were performed in a temperature range from −80 to 200 °C under N_2_ atmosphere, with a heating rate of 20 °C∙min^−1^.

#### 2.6.2. ATZ Quantification and Adsorption Kinetics Evaluation

Firstly, absorption spectra were obtained at different ATZ concentrations, from 1 to 11 mg∙L^−1^, in a Perkin Elmer Lambda 35 ultraviolet–visible light (UV–Vis, Metepec, Mexico) spectrophotometer, at a wavelength value of 222 nm. Subsequently, mixtures of 35 mg of raw clay mineral, OMH, OMP, or CCC samples, and 10 mL of ATZ solutions (5 mg∙L^−1^) were shaken in an orbital bath for different periods of time, from 4 to 24 h, at 25 °C and 100 rpm. Lastly, samples were decanted, and the supernatants were centrifuged at 1500 rpm for 20 min, and the concentration of ATZ in the supernatant was determined by means of the UV–Vis technique, following the procedure reported previously [16]. It is noteworthy that all adsorption kinetics experiments were performed at least in triplicate.

The *q*_e_ values (equilibrium adsorption capacity) were determined from the adsorption kinetics curve: once the *q*_t_ values did not change, we could assume that the equilibrium was reached; therefore, *q*_e_ = *q*_t_.

In addition, *q*_t_ was determined from the following equation:

*q*_t_ = [(*C*_i_ − *C*_t_)/*w*] V, (3)

where *C*_i_ is the initial concentration of the ATZ solution in mg·L^−1^, *C*_t_ is the ATZ concentration in a determined time t (mg·L^−1^), w is the mass of the dry adsorbent material (g), and V is the volume of the solution (L).

## 3. Results

### 3.1. Organo-Modification of the Raw Clay Mineral

#### 3.1.1. CEC

Table 2 summarizes the obtained CEC results for the raw clay mineral (sepiolite), as reported by the API [29]; this procedure was carried out in triplicate. Furthermore, to be more accurate, 0.1 and 0.5 g samples were weighed to compare the obtained results. The mineral presented a CEC of 29.6 mEq/100 g, which is in concordance with what is reported in the literature for a sepiolite [31,32,33].

#### 3.1.2. XRD Analysis

**Figure 2:**
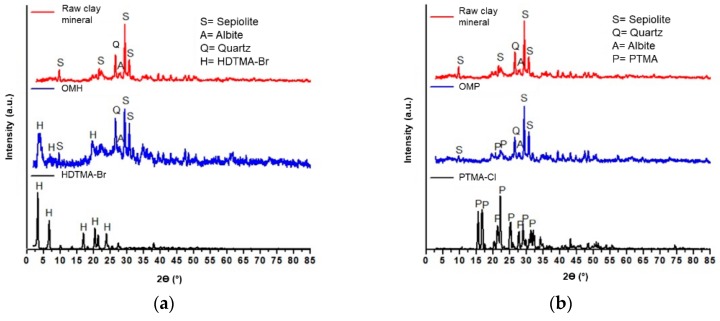
X-ray diffraction (XRD) patterns of the (**a**) raw clay mineral, the organo-modified with hexadecyltrimethylammonium bromide (OMH), and the hexadecyltrimethylammonium bromide (HDTMA-Br) cationic surfactant. (**b**) The raw clay mineral, the organo-modified with phenyltrimethylammonium chloride (OMP) and the phenyltrimethylammonium chloride (PTMA-Cl) cationic surfactant.

Figure 2a shows the XRD patterns of the raw clay mineral, the OMH, and the HDTMA-Br cationic surfactant. In the case of the first pattern, reflections corresponding to the main clay minerals present in the structure were identified and corresponded to sepiolite (JCPDS 00-023-0330), albite (JCPDS 00-001-0739), and quartz (JCPDS 00-005-0490). Moreover, the pattern corresponding to the HDTMA-Br surfactant was also observed, and it was compared to the JCPDS 00-030-1746 card. In general terms, it can be seen that the obtained XRD pattern maintained the same crystallographic structure of the HDTMA cationic surfactant. Finally, the pattern of the OMH presented a displacement to the right, in angles 2θ, compared to the raw clay mineral, which was a result of the decrease of the interlaminar space and is proof that a change in the crystalline structure of the material was carried out mainly in the interlaminar zone.

On the other side, the XRD patterns of the raw clay mineral, the OMP, and the PTMA-Cl cationic surfactant are shown in Figure 2b; however, a different behavior was observed. In this case, peaks did not present any shift and maintained the same pattern. Therefore, it is suggested that the exchange process of the PTMA cations within the clay structure was achieved mainly with the external cations present in the surface of the raw clay mineral [21].

### 3.2. Organo-Modified Clay Nanocomposite (CC) Characterization

#### 3.2.1. Swelling Behavior

**Figure 3:**
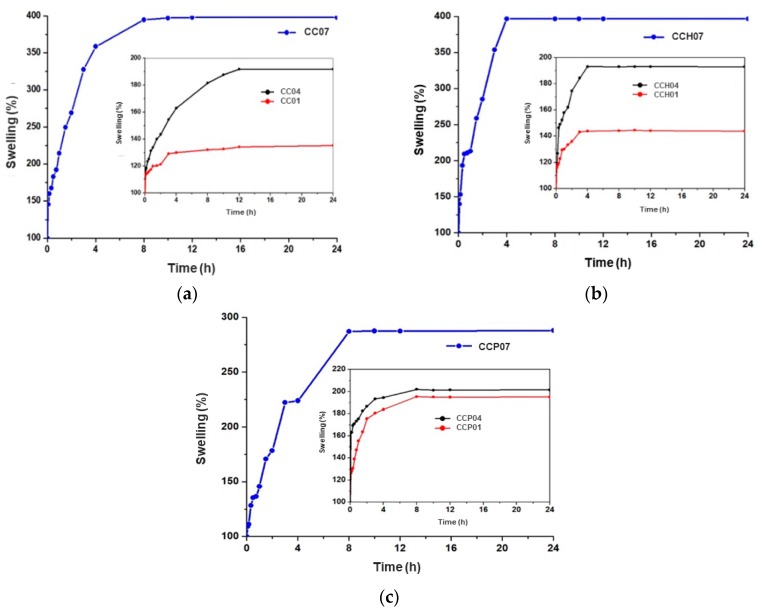
Equilibrium swelling time of different systems: (**a**) CC01, CC04, and CC07; (**b**) CCH01, CCH04, and CCH07 nanocomposites modified with HDTMA; (**c**) CCP01, CCP04, and CCP07 nanocomposites modified with PTMA.

Firstly, we studied the swelling properties of the synthesized materials. Figure 3a shows the swelling percentage of a synthesized p(4VP*-co-*AAm) copolymer, sample CC04, and the homopolymers p(4VP) and p(AAm), which correspond to samples CC01 and CC07, respectively. Sample CC07 reached its equilibrium after 8 h with a maximum swelling percentage of 400%. However, it showed poor mechanical properties when it was swollen, since its structure collapsed. Meanwhile, the sample CC01 reached its maximum swelling capacity at 12 h with a maximum percentage of 140%. Finally, the copolymer CC04 had its maximum swelling capacity at 12 h with a percentage of 190%; this decrease in the swelling capacity, compared to the homopolymer CC07, was mainly due to the presence of the 4VP moiety in the copolymer matrix, which is more hydrophobic than the AAm moiety.

Figure 3b shows the equilibrium swelling of nanocomposites with OMH, which occurred very quickly during the first 4 h for all the materials. In the case of nanocomposite CCH07, a maximum swelling percentage of 400% was found. On the other side, the nanocomposite CCH01 had a maximum swelling percentage of 140%. Finally, the nanocomposite CCH04 presented a maximum swelling percentage of 190%. It is important to mention that the swelling capacity between polymers and their nanocomposites with OMH did not vary significantly; however, the time in which the equilibrium was reached changed significantly, going from an equilibrium swelling time of 12 to 4 h.

At last, Figure 3c shows the swelling behavior for nanocomposites synthesized with OMP. For all synthesized materials, the equilibrium time was found after 8 h; in the case of nanocomposite CCP07, it reached a maximum percentage of 290%. Meanwhile, the nanocomposite CCP01 exhibited a maximum swelling percentage of 190%, and the nanocomposite CCP04 showed a swelling capacity of 200%.

**Figure 4:**
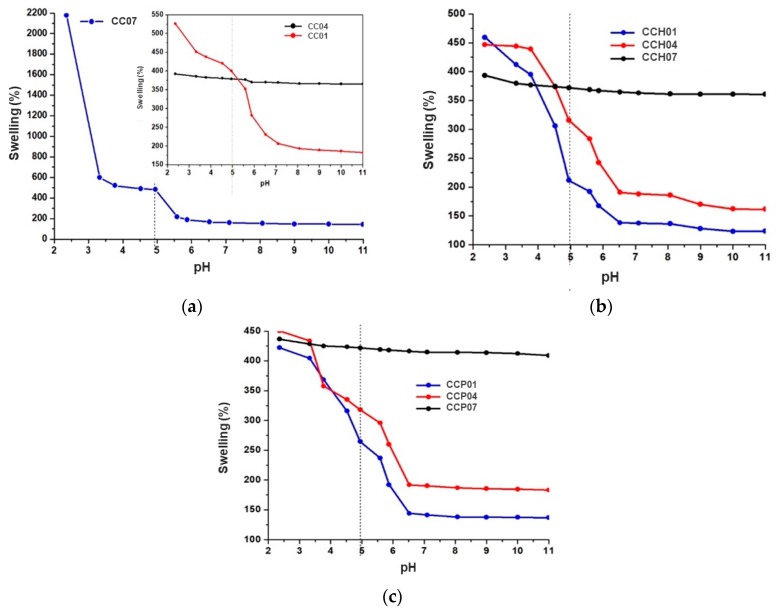
Critical pH value of different systems: (**a**) CC01, CC04, and CC07 copolymers; (**b**) CCH01, CCH04, and CCH07 nanocomposites modified with HDTMA; (**c**) CCP01, CCP04, and CCP07 nanocomposites modified with PTMA.

Figure 4a displays the critical pH value of the synthesized homopolymers and copolymer. For the homopolymer CC07, it was confirmed that the swelling capacity did not depend on the pH of the aqueous solution; therefore, it is a polymer that does not respond to the pH stimulus. In contrast, the homopolymer CC01 showed variable swelling capacities due to the basic nature of the pyridine nitrogen ring [34]. The critical pH value determined was found to be 4.9, which was very similar to that reported by Clara-Rahola et al. [35]. This homopolymer presented a significant swelling capacity, because, at these values, the pyridine groups of p(4VP) are protonated; thus, the repulsive electrostatic contribution induces the swelling of the polymer structure forming hydrogen bonds with the water molecules in the medium. Finally, the copolymer CC04 showed a very similar behavior to that of homopolymer CC01, with a critical pH value of 5.0; thus, it is understood that the behavior of this copolymer was significantly influenced by the p(4VP) moiety in its structure.

Figure 4b illustrates the critical pH value of nanocomposites synthesized with the organo-modified clay with HDTMA. From this figure, it can be seen that, for nanocomposite CCH01, the critical pH value did not show any significant difference compared to the homopolymer CC01 and it was practically the same, i.e., 4.9. Similarly, it was observed that the nanocomposite CCH04 did not show any significant difference with the copolymer CC04, with a critical pH value of 5.

Finally, Figure 4c represents the critical pH value of nanocomposites synthesized with the organo-modified clay with PTMA. From this figure, it can be observed that the critical pH values for nanocomposites with this organo-modified clay had a similar behavior to the synthesized nanocomposites with HDTMA, i.e., pH = 4.9.

**Figure 5:**
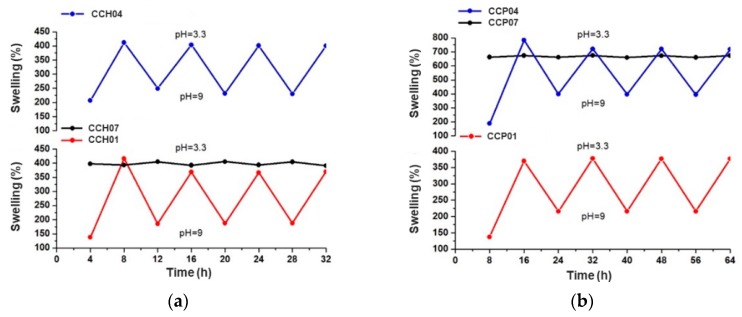
pH reversibility for different systems: (**a**) CCH01, CCH04, and CCH07 nanocomposites modified with HDTMA; (**b**) CCP01, CCP04, and CCP07 nanocomposites modified with PTMA.

Figure 5a,b show the reversibility of the extended and collapsed states for the synthesized p(4VP-*co*-AAm) nanocomposites with the organo-modified clays HDTMA and PTMA, respectively. Measurements were performed below and above the critical pH value, that is, at pH = 3.3 and pH = 9, after swelling for 4 or 8 h, depending on the nanocomposite sample. In the case of Figure 5a, nanocomposites CCH01 and CCH04 showed very good reversibility response with pH sensitivity, S_w9_/S_w3.3_ = 2.56. In the case of nanocomposite CCH07, since AAm did not have a response to pH, it did not demonstrate any response to this external stimulus. Figure 5b corresponds to nanocomposites CCP01, CCP04, and CCP07. Similarly, these materials displayed a pH sensitivity value, S_w9_/S_w3.3_ = 2.54, which is in concordance with that obtained for nanocomposites modified with HDTMA.

#### 3.2.2. FTIR

**Figure 6:**
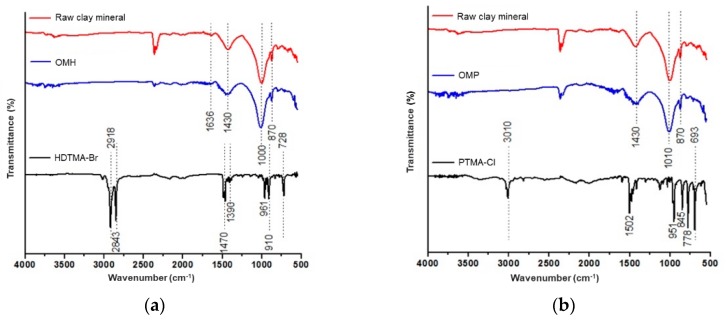
Fourier-transform infrared (FTIR) spectra of (**a**) raw clay mineral, OMH, and HDTMA-Br cationic surfactant; (**b**) raw clay mineral, OMP, and PTMA-Cl cationic surfactant.

Figure 6a shows FTIR spectra corresponding to raw clay mineral, OMH, and HDTMA cationic surfactant. In this case, three important signals were observed that confirmed the OMH organo-modification with HDTMA; namely, the first signals between 2918 and 2843 cm^−1^ were due to the stretching vibrations of the –CH_2_ groups present in the hydrocarbon chain, and the third signal at 1470 cm^−1^ corresponded to the bending vibration of this same bond. In addition, the increase in the intensity of all these signals was related to the amount of ammonium salt used to obtain this organo-modified clay. In the case of Figure 6b, the most important signal representative of the organo-modification with PTMA was found at 1502 cm^−1^, which could be attributed to the bending vibrations of the C–N bond of the quaternary ammonium salt.

**Figure 7:**
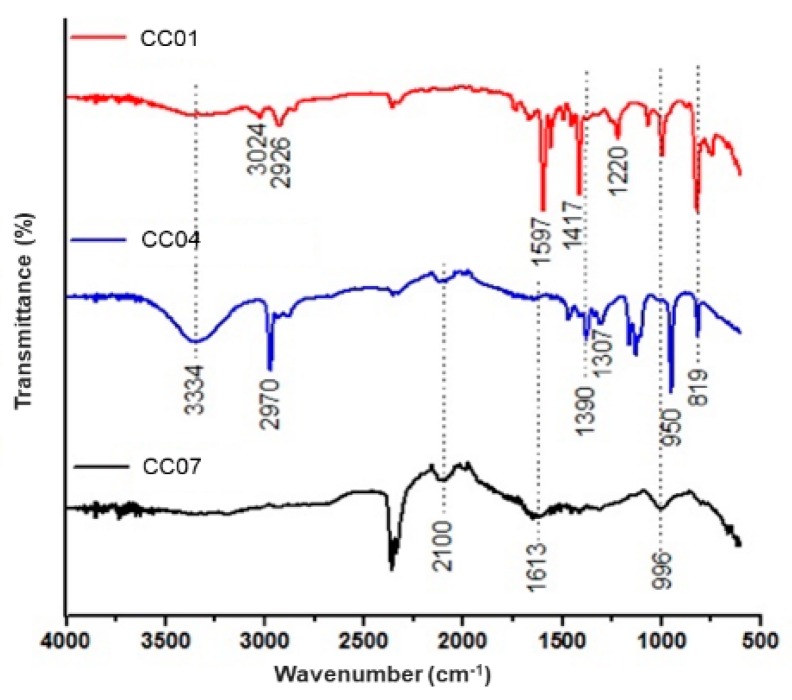
FTIR spectra of CC01, CC04, and CC07 copolymer matrices.

Figure 7 shows the FTIR spectra of samples CC01, CC04, and CC07. In the case of homopolymer CC01, the absorption bands located between 1600 and 1419 cm^−1^ corresponded to the stretching vibrations of the pyridine ring, while the band at 821 cm^−1^ was associated with the symmetric vibrations of the monosubstituted ring of pyridine. Moreover, bands at 1492 and 1451 cm^−1^ were characteristic of the C–C stretching vibrations of the benzyl ring and the stretching vibrations of the C–H bond of this same ring, respectively. Finally, other bands in this spectrum were associated with the stretching vibration of the aliphatic CH_2_, at 2914 cm^−1^, and aromatic CH, at 3026 cm^−1^, and the medium intensity bands, located in the range between 1250 and 1000 cm^−1^, with C=N bonds.

Meanwhile, in the spectrum corresponding to the copolymer CC04, the characteristic bands of p(4VP) could be observed at 2914 cm^−1^, associated with the stretching vibration of the aliphatic CH_2_ bond, and at 821 cm^−1^, corresponding to the symmetric vibrations of the monosubstituted pyridine ring. In the same way, the bands corresponding to acrylamide were found at 2100 cm^−1^ which corresponds to the N–H combination of stretching and torsional vibrations, and at 1613 cm^−1^, which corresponds to the stretching of the C=O bond of the acrylamide.

Lastly, in the case of the homopolymer CC07, two characteristic bands corresponding to the N–H bond were observed; the first one was located at 3359 cm^−1^, which was due to its asymmetric stretching vibrations, and the second one was at 3203 cm^−1^, due to its symmetric stretching vibrations. In addition, between 2914 and 2800 cm^−1^, vibrations of the CH_2_ bonds of the aliphatic chain were observed. Finally, the absorption band at 1684 cm^−1^ indicated the stretching vibrations of the carbonyl group, C=O, corresponding to the primary amides of acrylamide.

#### 3.2.3. SEM

**Figure 8:**
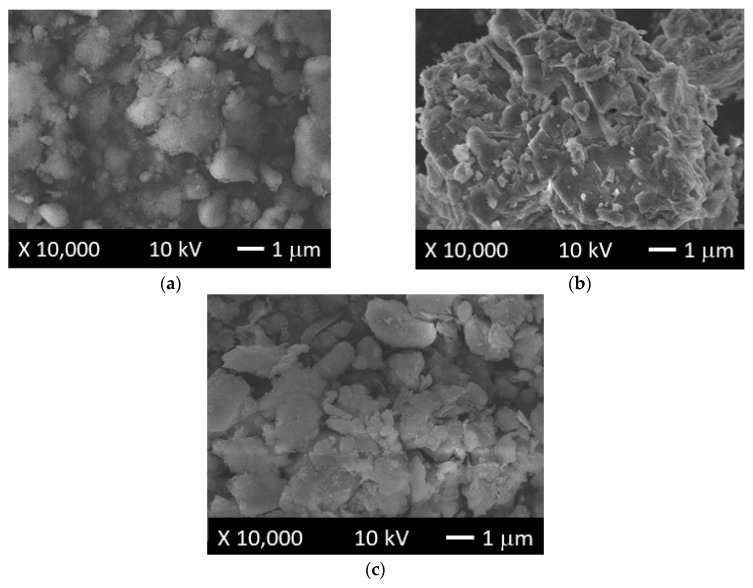
SEM micrographs of (**a**) raw clay mineral (sepiolite), and (**b**) OMH- and (**c**) OMP-modified clays.

Figure 8a–c show micrographs of the raw clay mineral (sepiolite), OMH, and OMP organo-modified clays, respectively. In Figure 8b, the OMH surface showed agglomerates of some particles, which were not identified on the surface of sepiolite, depicted in Figure 8a. The same behavior was observed in Figure 8c, which corresponds to the OMP organo-modified clay, where a more homogeneous surface could be observed, with respect to that of sepiolite. These changes could be attributed to the presence of the cationic surfactants that were not present in the structure of the sepiolite, which is in concordance with a previous report concerning the organo-modification of different types of clays [36,37].

**Figure 9:**
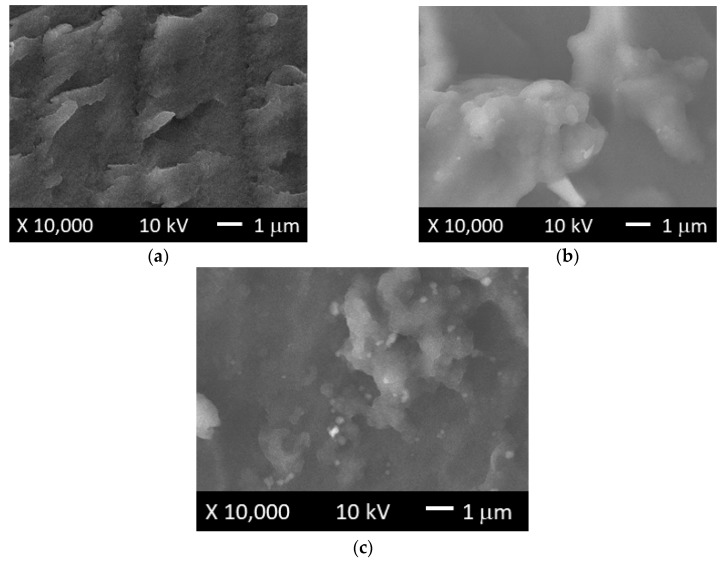
SEM micrographs of (**a**) CC04 copolymer, and (**b**) CCH04 and (**c**) CCP04 nanocomposites.

In the case of Figure 9a–c, SEM micrographs of copolymer CC04, and nanocomposites CCH04 and CCP04, respectively, are shown. As it can be seen, copolymer CC04 had a more regular surface; in comparison, nanocomposites CCH04 or CCP04 exhibited a more heterogeneous surface with some little aggregates randomly distributed onto their surface. This change could be attributed to the incorporation of the OMH or OMP into the structure of the material that was not present in the sample CC04.

#### 3.2.4. Thermal Analysis

TGA and DSC analyses (not shown) of the raw clay mineral (sepiolite), the organo-modified clays (OMH and OMP), and CC04, CCH04, and CCP04 polymer and nanocomposite samples were performed and are summarized in Table 3.

In the case of the TGA analyses, the thermal stability of clay materials was very good, and, for all materials, the obtained *T*_5_ values (i.e., the temperature at which the sample loses 5% mass) were found between 480 to 520 °C depending on the nature of the raw clay mineral or the employed cationic surfactant for its organo-modification. As for the copolymer or nanocomposite samples, these values were found from 140 to 150 °C; however, this stability decreased when the organo-modified clay was incorporated into the copolymer matrix structure, which is in concordance with some previous reports [18,21].

Meanwhile, from the DSC analyses, it was found that *T*_g_ values for copolymer and nanocomposite samples were around 165 to 168 °C, depending on the incorporated organo-modified clay into its structure. This slight change could be attributed to the plasticizer effect that clay minerals could provide to the polymer matrix, since they have an organophilic nature due to their organo-modification with the HDTMA or PTMA cationic surfactants.

### 3.3. Adsorption Experiments

#### 3.3.1. Adsorption Kinetics of the Synthesized Nanocomposites with the CC04 Copolymer

Adsorption kinetics allows evaluating the potentiality of a material to be used as an adsorbent. Therefore, it is important to determine the time for which the adsorption process reaches its equilibrium, as well as the kinetics parameters, to understand the behavior of the materials in the atrazine adsorption process.

**Figure 10:**
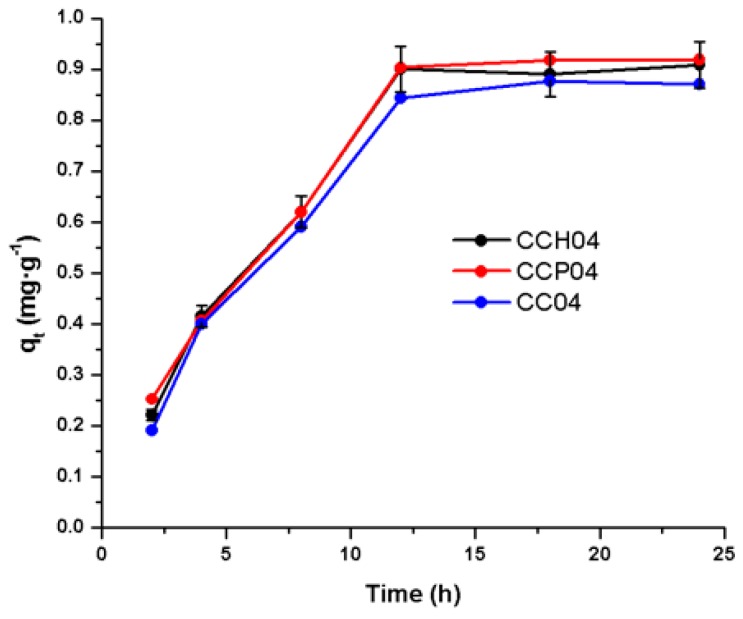
Adsorption kinetics for the synthesized materials: CC04 copolymer, and CCH04 and CCP04 nanocomposites.

Figure 10 represents the adsorption kinetics for the CC04 and respective nanocomposites, i.e., CCH04 and CCP04. From this figure, it can be observed that all the synthesized materials presented a maximum adsorption capacity at 12 h, with ATZ removal being slightly higher in the CCP04 nanocomposite. A similar behavior was obtained for the OMH and OMP (not shown).

It is noteworthy that, when all materials reached the equilibrium time (12 h), they maintained a constant adsorption capacity; this indicates that the copolymer matrix contributed to keeping the ATZ molecule stable on the surface of the material during the adsorption process. On the other hand, from this same figure, it can be seen that nanocomposites CCP04 and CCH04 presented *q*_t_ values slightly higher with respect to the CC04 copolymer, since the incorporation of the organo-modified clays into the copolymer matrix conferred synergic properties to the nanocomposite materials.

**Figure 11:**
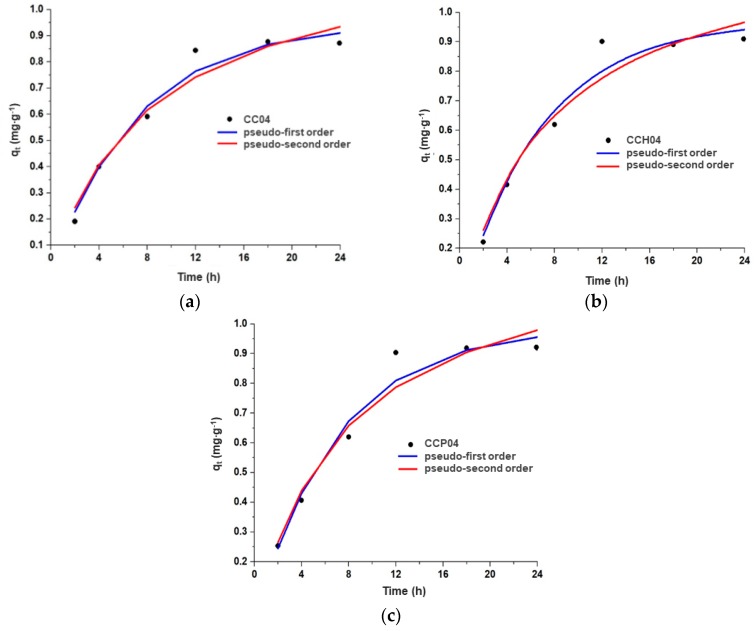
Kinetics models of the different synthesized materials: (**a**) CC04 copolymer, and (**b**) CCH04 and (**c**) CCP04 nanocomposites.

Moreover, in order to know the nature of the system, the experimental data were associated with the following kinetics models: pseudo-first-order and pseudo-second-order, as depicted in Figure 11a–c. Comparing the two proposed models for the ATZ adsorption, it is observed in Table 4 that all the synthesized materials presented a better fit with the pseudo-first-order model, obtaining higher values of the correlation coefficient (*R^2^*) very close to 1, and lower values of chi-square (χ^2^), indicating that the limiting step for the ATZ adsorption process was the mass transfer of the herbicide from the solution to the surface of the adsorbent [38]. In the case of the value of the constant K, it was observed that nanocomposites CCH04 and CCP04 presented higher values than the CC04 copolymer, which was an indication that the adsorption speed of ATZ was higher; consequently, the value of constant K depended on the content of the quaternary ammonium salt present in the nanocomposite.

Therefore, from these results, it can be observed that the pseudo-first-order model is the one that was best fitted to the experimental data, which is an indication that the mechanism of physisorption was predominant in the adsorption process.

#### 3.3.2. Effect of the 4VP and AAm Molar Fractions in the Obtained Nanocomposites

**Figure 12:**
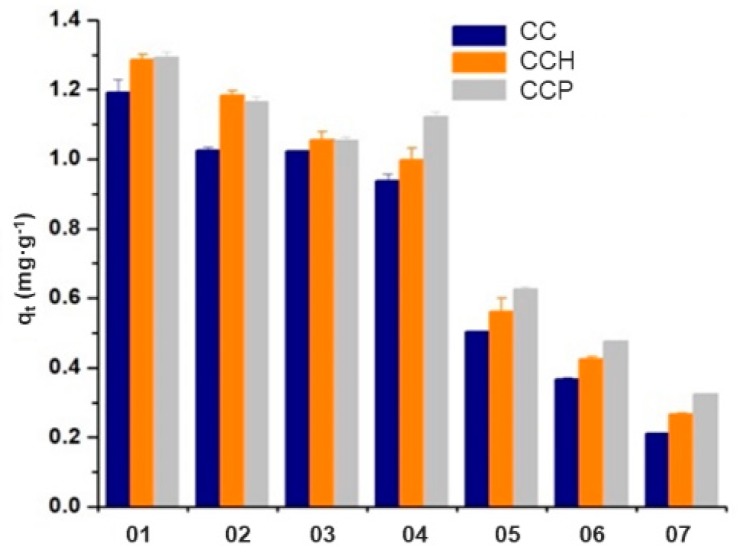
Removal capacities of the synthesized copolymers, with different of 4-vinylpyridine (4VP)/ acrylamide (AAm) co-monomer ratios in their structures.

Figure 12 presents the contact tests that were carried out for the polymeric matrix, and the nanocomposites with HDTMA or PTMA in all the stoichiometric ratios considered for the synthesis. Briefly, 0.0367 g of each material was put in contact with 10 mL of a commercial ATZ solution with an initial concentration of 5 mg∙L^−1^; all tests were carried out at 25 °C, 100 rpm, with an equilibrium time of 12 h. After this period of time, samples were decanted to separate the aqueous phase from the adsorbent material. From this figure, it can be observed that from the synthesized materials, the p(4VP) homopolymer, and its nanocomposites, with HDTMA and PTMA organo-modified clays, showed the highest adsorption capacities, with *q*_t_ values of 1.192 mg∙g^−1^ for the homopolymer, and between 1.286 and 1.293 mg∙g^−1^ for the nanocomposites with HDTMA of PTMA, respectively.

Moreover, it was also observed that, when the ratio of acrylamide in the copolymer and nanocomposite structures increased, the adsorption capacity of ATZ with these materials decreased. Nevertheless, in the case of copolymers or nanocomposites with 85:15, 65:35, and 50:50 ratios between 4VP and AAm, the ATZ removal capacity was very similar, i.e., around 15%, which is in agreement with the data previously reported by Gardi et al. [5]. Finally, nanocomposites with PTMA organo-modified clay into their structure were more efficient than HDTMA nanocomposites, which can probably be attributed to the cationic surfactant structure that has an aromatic ring in its chemical structure. Table 5 shows the percentages of the synthesized copolymers, with different molar fractions of 4VP and AAm in their structures.

#### 3.3.3. Effect of the Mass–Volume Ratio on the Capacity to Remove ATZ with the Modified and Synthesized Materials

**Figure 13:**
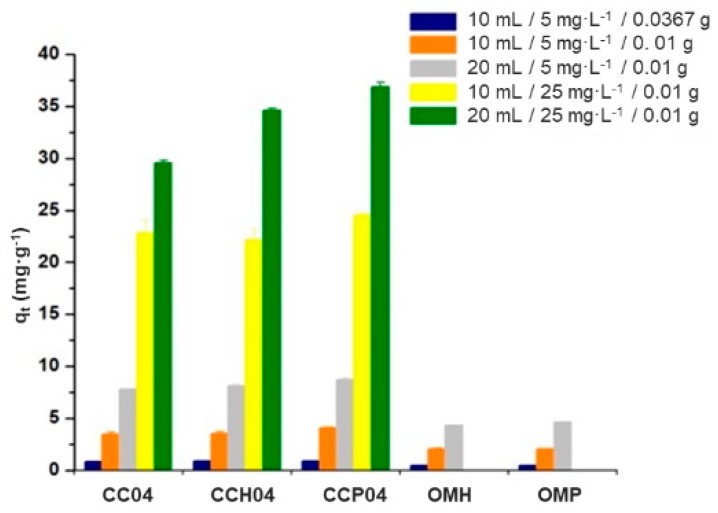
Adsorption experiments varying the mass–volume ratio and the initial concentration of atrazine in the prepared solutions.

Finally, the relationship between the mass of the adsorbent and the initial concentration of ATZ in the solution was evaluated in this work, and the results are presented in Figure 13. In the first case, an unexpected behavior, i.e., a decrement of the mass of the adsorbent material, from 0.0367 to 0.01 g, favored the ATZ adsorption process with both copolymers and nanocomposites, with an increase in the adsorption capacity of 400%. As for the effect of the initial concentration of ATZ on its adsorption process, when the concentration increased from 5 to 20 mg∙L^−1^ in the aqueous solutions, it was observed that the adsorbed amount of the herbicide with materials was favored, until it reached six times its initial removal capacity. It is worth noticing that this was only observed in the polymeric materials; on the contrary, the organo-modified clays did not show this behavior, since the active sites were occupied quickly by the herbicide, causing saturation of the material. Lastly, the effect of the volume of solutions on the ATZ adsorption capacity was evaluated, and it was observed that this parameter was strongly associated with the initial concentration of the herbicide, since, at low ATZ concentrations (5 mg∙L^−1^), the adsorption capacity was 200%; however, when the concentration of the analyte was increased to 25 mg∙L^−1^, the adsorption capacity diminished to only 50%. Therefore, the effect of the volume on the adsorption capacity was lesser compared to the effects of variations of the adsorbent mass and initial ATZ concentration.

## 4. Discussion

From these results, it was demonstrated that p(4VP*-co-*AAm) copolymers and their nanocomposites with an organo-modified clay were obtained through the radical polymerization technique. Firstly, the nature of the hydrophilic raw clay mineral was effectively changed to a hydrophobic one, with the incorporation of two different types of organo cationic surfactants, HDTMA of PTMA. From the obtained XRD patterns, it is important to remark that the expansion of the interlaminar spaces could be an indication of the presence of the surfactant molecules in the organo-modified clay and this became evident because of the shift of the peaks toward lower angles in 2θ, as well as the appearance of new ones.

When the percentage of hydrophilic monomer (AAm) in the synthesis of the copolymers was increased, the maximum swelling percentage at the equilibrium was augmented, and the absorption time of water decreased by action of the ionic forces between the functional groups of the copolymer chain and the surrounding medium. This decrement in the equilibrium time was attributed to the incorporation of the organo-modified clay within the polymer matrices, and the swelling capacity was similar between nanocomposites and these matrices, due to the hydrophobic nature of these organo-modified clays. Furthermore, it was observed that, at pH values below the critical pH value, the swelling capacity decreased in comparison with the polymer matrices; however, it was also lower compared to the nanocomposite with the HDTMA-modified clay, because the organo-modification of the clay with the PTMA surfactant and the type of structure that it presented prevented the penetration of water inside the matrices; therefore, a decrement in the swelling capacity was observed. Furthermore, all of the synthesized materials confirmed an excellent response to cyclical changes in pH, demonstrated by their stable sensitivity to pH value.

In the case of FTIR spectra, the successful incorporation of the organo-modified clays into the polymer matrices was confirmed by the appearance of bands related to silicates and the alkyl chains of both cationic surfactants. In addition, according to the TGA, the amount of absorbed water decreased when the organo-modified clays with HDTMA or PTMA were incorporated into the matrix structures. Moreover, the thermal stability of nanocomposites was higher than that of the polymer matrices due to the incorporation of these organo-modified clays in their structures. Specifically, the value of *T*_g_ for the p(4VP) differed from that reported in the literature probably due to the amount of cross-linking agent used in the synthesis of the polymers, which gave them a higher plasticizer effect.

The kinetics results indicated that nanocomposites reached their maximum adsorption capacity from 12 up to 24 h. This indicates that the copolymer matrix contribution was the stability of the ATZ molecule on the surface of the material during the adsorption process. The kinetics model that best fitted the experimental data was the pseudo-first-order model, which pointed out that the mechanism of physisorption was predominant during the adsorption process. Finally, the relationship between mass and volume with the initial concentration of ATZ played an important part in the removal capacity of the obtained materials, and it is an important consideration for future applications.

## 5. Conclusions

In this work, two different series of copolymers and their nanocomposites, obtained from 4VP and AAm with the incorporation of two diverse organo-modified clays, were successfully synthesized. It was confirmed that the swelling capacity of the synthesized materials was influenced by the percentage of the hydrophilic monomer (AAm) into their structure; i.e., the higher the hydrophobic monomer (4-VP) moiety, the lower the swelling capacity of the materials. The critical pH value (pH = 4.9) did not vary between the polymer matrix and the synthesized nanocomposites because of the amount of the organo-modified clay that was dispersed within the polymer matrix. The pH sensitivity test showed that the physical properties of the copolymers were not affected by the incorporation of the organo-modified clay within its structure, since this dispersion was very low. As it was previously confirmed, the thermal stability of the obtained nanocomposites was greater than that of the copolymers due to the incorporation of the organo-modified clay into their structure. Lastly, from the adsorption kinetics experiments, it was demonstrated that the modification of the raw clay mineral with the HDTMA-Br and PTMA-Cl surfactants improved its performance for the ATZ removal; and the kinetics model of pseudo first order was the one that best described the ATZ sorption from aqueous solutions.

## Figures and Tables

**Table 1:** Experimental data of the synthesized samples. 4VP—4-vinylpyridine; AAm—acrylamide; OMH—organo-modified with hexadecyltrimethylammonium bromide; OMP—organo-modified with phenyltrimethylammonium chloride; BIS *N*,*N*’-methylenebis(acrylamide).

Sample Codes	4VP (mol. %)	AAm (mol. %)	OMH/OMP (wt. %)	BIS (mol. %)	TBPPS (mol. %)
CC01	100	0	-	3	0.25
CC02	85	15	-	3	0.25
CC03	65	35	-	3	0.25
CC04	50	50	-	3	0.25
CC05	30	70	-	3	0.25
CC06	10	90	-	3	0.25
CC07	0	100	-	3	0.25
CCH01	100	0	5	3	0.25
CCH02	85	15	5	3	0.25
CCH03	65	35	5	3	0.25
CCH04	50	50	5	3	0.25
CCH05	30	70	5	3	0.25
CCH06	10	90	5	3	0.25
CCH07	0	100	5	3	0.25
CCP01	100	0	5	3	0.25
CCP02	85	15	5	3	0.25
CCP03	65	35	5	3	0.25
CCP04	50	50	5	3	0.25
CCP05	30	70	5	3	0.25
CCP06	10	90	5	3	0.25
CCP07	0	100	5	3	0.25

**Table 2:** Cationic exchange capacity (CEC) results for the raw clay mineral.

Raw Clay Mineral (g)	Volume of Methylene Blue (mL)	CEC (meq/100 g)
0.5006	15	29.96
0.5003	15	29.86
0.4996	14	28.02
0.1006	3	29.82
0.1004	3	29.88
0.1002	3	29.94

**Table 3:** Thermogravimetric analysis (TGA) results of the employed materials.

Sample Code	*T*_5_ (°C)	*T*_g_ (°C)
Raw clay mineral	500	ND ^1^
OMH	480	ND ^1^
OMP	520	ND ^1^
CC04	150	165
CCH04	140	167
CCP04	140	168

^1^ ND = not determined.

**Table 4:** Parameters for the kinetics models of the synthesized materials.

Material	*q*_e_(exp) (mg∙g^−1^)	Pseudo-First-Order Model			Pseudo-Second-Order Model		
		*K*_L_ (h^−1^)	*q*_e_ (mg∙g^−1^)	*R^2^*	*K*_2_ (g∙mg^−1^·h^−1^)	*q*_e_ (mg∙g^−1^)	*R^2^*
**CC04**	0.845	0.137	0.945	0.986	0.095	1.258	0.978
CCH04	0.900	144	0.972	0.983	0.100	1.278	0.974
CCP04	0.903	143	0.988	0.984	0.098	1.298	0.976

**Table 5:** Removal percentages and q average value for the different synthesized materials.

Material	Copolymer (CC)		HDTMA Nanocomposites (CCH)		PTMA Nanocomposites (CCP)	
	Removal (%)	*q*_t_ Average (mg∙g^−1^)	Removal (%)	*q*_t_ Average (mg∙g^−1^)	Removal (%)	*q*_t_ Average (mg∙g^−1^)
01	92	1.192 ± 0.036	94	1.286 ± 0.015	95	1.293 ± 0.014
	92		94		95	
02	81	1.026 ± 0.007	82	1.185 ± 0.011	83	1.165 ± 0.015
	81		82		83	
03	76	1.024 ± 0.002	79	1.056 ± 0.023	80	1.054 ± 0.009
	75		78		80	
04	71	0.938 ± 0.019	73	0.998 ± 0.033	78	1.122 ± 0.015
	69		73		78	
05	37	0.505 ± 0.002	39	0.562 ± 0.038	44	0.625 ± 0.007
	36		41		46	
06	28	0.366 ± 0.005	31	0.425 ± 0.007	35	0.476 ± 0.001
	27		31		36	
07	15	0.210 ± 0001	19	0.266 ± 0.003	22	0.324 ± 0.002
	16		21		24	
